# The American Homœopathic Pharmacopœia

**Published:** 1883-10

**Authors:** 


### BOOK NOTICES AND REVIEWS.

The American Homœopathic Pharmacopœia, second edition. Thoroughly revised and augmented. Pp. 510. Edited by Dr. J. T. O'Connor. New York and Philadelphia: Bœricke & Tafel, 1883.

The first edition of this work appeared some months ago. It contains some botanical, pharmaceutical, or chemical data taken from the dispensatory or pharmacopœia of the allopath. Immediately our *liberal-minded, generous friends* yelled, "Stop thief!" They had been long accustomed to appropriate our useful discoveries; but for us to utilize some of their well-known, old-time data it was a crime in their eyes! They say we homœopaths are ignorant, and they don't intend for us to be wise *unless* we *buy* wisdom of them. However, we can thank them for their ill-advised malice, since it has brought us this *second edition* of the *American Homœopathic Pharmacopœia.* Dr. O'Connor has improved it very much. And now that we have such a standard, let us prepare our drugs properly and spell them correctly.

